# Effects of Transcranial Direct Current Stimulation on Brain Electrical Activity, Heart Rate Variability, and Dual-Task Performance in Healthy and Fibromyalgia Women: A Study Protocol

**DOI:** 10.3390/bs12020037

**Published:** 2022-02-04

**Authors:** Mari Carmen Gomez-Alvaro, Santos Villafaina, Juan Luis Leon-Llamas, Alvaro Murillo-Garcia, María Melo-Alonso, Jesús Sánchez-Gómez, Pablo Molero, Ricardo Cano-Plasencia, Narcis Gusi

**Affiliations:** 1 Physical Activity and Quality of Life Research Group (AFYCAV), Faculty of Sport Sciences, University of Extremadura, 10003 Cáceres, Spain; maricarmengomezal@unex.es (M.C.G.-A.); svillafaina@unex.es (S.V.); alvaromurillo@unex.es (A.M.-G.); meloalonsomaria@gmail.com (M.M.-A.); jesusssg6@gmail.com (J.S.-G.); pmolero@unex.es (P.M.); ngusi@unex.es (N.G.); 2 Clinical Neurophysiology, San Pedro de Alcántara Hospital, 10003 Cáceres, Spain; ricanopla@hotmail.com; 3 International Institute for Innovation in Aging, University of Extremadura, 10003 Cáceres, Spain

**Keywords:** tDCS, chronic pain, EEG, activities of daily living, prefrontal cortex, postural balance, muscle strength, creativity

## Abstract

People with fibromyalgia could experience physical and cognitive impairments. Furthermore, when performing two tasks at the same time, people with fibromyalgia showed a higher dual-task cost compared to a single task than healthy people. This may result in poorer performance of activities of daily living that are commonly presented as a combination of two or more tasks. Transcranial direct current stimulation (tDCS) is a promising nonpharmacological therapy. However, there is controversy regarding the intensities and the effectiveness of this therapy. Thus, the present study will aim: (1) to compare the effectiveness and the impact of two tDCS intensities (1 mA and 2 mA) on cognitive, motor, brain functions, and cardiac autonomic modulation; (2) to study the impact of tDCS on the dual-task performance and creativity after applying tDCS in dorsolateral prefrontal cortex. In this study, 26 women will participate and will be divided into two groups: women with fibromyalgia (*n* = 13), and healthy controls (*n* = 13). A reduction in cognitive-motor interference in dual-task performance is expected, as well as a modification in neurophysiological parameters and an improvement in cardiac autonomic modulation. Lastly, no different effects are expected depending on the stimulation intensity applied. The obtained results will help to determine if tDCS in the dorsolateral prefrontal cortex could improve the occupational performance of women with fibromyalgia.

## 1. Introduction

Fibromyalgia (FM) is characterized by widespread pain and other associated symptoms, including stiffness, fatigue, nonrecovery sleep, anxiety, or depression [1]. On a physical level, people with FM frequently suffer from a sedentary state which reduces cardiovascular fitness and leads to mobility, strength, and balance impairments [2,3,4]. On a cognitive level, people with FM have often impaired cognitive functions such as memory, attention, processing speed, and executive functions [5,6,7]. Furthermore, people with FM have shown an altered brain activity at rest [8,9] and autonomic modulation [10]. As a result of all of these symptoms and their consequences, people with FM have a reduced quality of life [11] and a decreased performance in activities of daily living [12].

Activities of daily living are commonly presented as a combination of two or more tasks at the same time [13,14]. This simultaneous task execution (motor–cognitive, cognitive–cognitive, or motor–motor) is known as the dual-task (DT) paradigm [15]. Previous research has shown that DT leads to an interference that causes a reduction in the performance of one or both tasks [16,17,18]. Regarding people with FM, a decrease in physical performance when two tasks are proposed simultaneously [19,20,21,22] has been observed. The prefrontal cortex (PFC), and particularly the lateral prefrontal cortex (LPFC) are the brain regions for which most interest has been generated, since this structure (LPFC) is mainly involved in coordination, organization, and prioritization processes in DT situations [16,23,24,25,26,27]. 

Different types of pharmacological and nonpharmacological therapies have been used to improve some FM symptoms [28]. Among the nonpharmacological therapies, one alternative therapy of interest is electrical therapy. In this regard, transcranial direct current stimulation (tDCS) is a noninvasive technique that modulates the excitability of the cortex by applying a low-intensity current [29]. This therapy appears to show changes in cortical excitability which persist for one hour when intensities range between 1 to 1.5 mA and the duration is greater 10 min [30]. The methods to apply this technique can be performed during the execution (online) or before the execution of the task (offline), measuring the direct or short/long term effects, respectively [31]. There is currently controversy about the most appropriate type of intensity, since previous studies have compared different intensities showing similar results [32,33,34,35,36,37,38,39]. In addition, higher intensities do not necessarily result in better effects [40,41,42,43]. Moreover, most of the studies have been focused on the primary motor cortex (M1). However, research focused on dorsolateral prefrontal cortex (DLPFC) stimulation has shown benefits in both young [27,44,45,46,47] and healthy older adults [48,49] by reducing the cost and improving the performance of DT.

In FM, the use of tDCS has been mainly focused on treating pain, using anodal stimulation in the motor cortex (M1) [50]. However, fewer studies have focused on applying tDCS to the PFC area and specifically on the DLPFC [51,52,53,54]. On the one hand, these studies have aimed to treat pain and sleep, showing decreased pain but no changes in sleep [51,52,53]. On the other hand, the application of PFC tDCS has been investigated to improve cognitive functions, reporting promising results in short- and long-term episodic memory and executive functions when combined with working memory training [54].

To our knowledge, no studies have focused on the application of tDCS on the DLPFC in people with FM to reduce interference in DT performance. Furthermore, there are no studies that have looked at the effects of different intensities of tDCS on PFC in people with FM in DT performance. Similarly, there are no studies that have focused on describing how neurophysiological variables may be modified in people with FM during DT performance. Therefore, it seems interesting to know how tDCS can influence these types of variables in order to improve the quality of life of this population and to open new frontiers for research.

### Hypothesis and Objectives

The hypotheses that are proposed are as follows: (a) the application of tDCS will improve physical performance in physical variables that will be measured through balance and strength tasks; (b) the application of tDCS will improve performance on neurophysiological variables that will be measured through brain electrical activity and HRV; (c) the application of tDCS will improve performance in DT conditions as well as in creativity tasks; (d) tDCS will have different effects depending on the variability of fibromyalgia symptoms presented (pain, sleep problems and depression; (e) no significant effects on the variables would be expected depending on the type of tDCS intensity applied.

At this point, the main objectives of this study are: (1) to compare the effectiveness and the impact of two tDCS intensities (1 mA and 2 mA) on neurophysiological variables, cognitive and motor functions; (2) to study the impact of tDCS on the dual-task performance and creativity after applying tDCS in the dorsolateral prefrontal cortex.

## 2. Materials and Methods

### 2.1. Study Design

This study is a double-blind, sham-controlled, crossover study. All participants will randomly participate through all conditions (1 mA, 2 mA, and sham). This study, in order to fulfil correctly the items of the standard protocol for clinical trials, has followed the SPIRIT 2013 Statement Items [55].

### 2.2. Participants

#### 2.2.1. Eligibility Criteria 

Participants will have to meet the following inclusion criteria to be part of the study: (a) female between 30–75 years of age; (b) be able to communicate with the research staff; (c) have read, understood, and signed the informed consent form. In addition, women with FM must have been diagnosed by a rheumatologist, according to the American College of Rheumatology criteria [1]. The exclusion criteria will be: (a) psychiatric or neurological disorders; (b) pharmacological treatment for anxiety or depression; (c) substance abuse or dependence; (d) contraindication for physical effort; (e) difficulty maintaining balance; (f) leg injury that interferes with flexion and extension of the knee; and (g) being pregnant.

#### 2.2.2. Recruitment

Taking into account the results of a previous investigation [56], a sample size of 13 achieves 99% power to detect a mean of paired differences of 3.5 with an estimated standard deviation of differences of 2.5 and with a significance level (alpha) of 0.05 using a two-sided Wilcoxon test assuming that the actual distribution is normal. 

Therefore, the sample will be collected during January/February 2022. One group will be composed of women with FM (*n* = 13) from the local association of persons with fibromyalgia, named AFIBROEX, while the other group will be composed of healthy women (*n* = 13).

#### 2.2.3. Randomization

Tasks and conditions will be randomized to avoid familiarization or learning effects. Randomization will be conducted using a random number generator (Random Number Generator tool; Google, LLC., Mountain View, CA, USA). Firstly, the order of application of the tDCS intensities on the three study days (1 mA, 2 mA, and sham) will be randomized. Secondly, the order of the execution of the tasks in the different tests will be randomized: balance (eyes open (EO), eyes closed (EC), EO-DT, EC-DT); strength (isokinetic dynamometry (ID), ID-DT). Thirdly, subtractions from an even or odd number will also be randomized.

### 2.3. Ethical Approval

The study protocol has been approved by the Research Ethics Committee of the University of Extremadura (192/2021).

### 2.4. Intervention

The transcranial direct current stimulation (tDCS) will be conducted with an 8-channel hybrid EEG/tDCS wireless neurostimulator (Starstim, NEuroelectrics, Barcelona, Spain). The electrodes used will be sponge electrodes (Sponstim^®^, 5.65 cm de diameter, 25 cm^2^ surface area) soaked in saline solution. 

Since the DT involves the prefrontal cortex in the main role [23], the active electrode will be placed on the left prefrontal cortex, corresponding to the F3 region of the international 10–20 electrode placement system. The return electrode will be placed on the contralateral supraorbital region (Fp2) [27,44,45,46,48,49,57,58]. At the same time, the HRV and EEG signals will be recorded at Cz, C4, P3, P4, and Oz. Patients will receive tDCS at an intensity of 1 mA (Condition 1) or 2 mA (Condition 2) for 20 min, with a ramp up and ramp down of 30 s each. A duration of 20 min will be applied to ensure that the acute effects remain for at least 1 h [30]. For the placebo or sham condition (Condition 3), the mode included in the NEuroelectrics NIC1 program will be used, consisting of a double sham with a 30 s double ramp up and ramp down. These parameters for Condition 3 were chosen based on previous reports of perceived skin sensations, which generally disappear within the first 30 s [59,60]. 

There will be a minimum of seven days between conditions. One stimulation session will be applied each week. A different stimulation will be applied each week. In total, three weeks are estimated for each participant to complete the study. The intervention and all assessments will be carried out at the Faculty of Sport Sciences (Caceres, Spain).

### 2.5. Procedures and Outcome Measures

#### 2.5.1. Procedure

**Figure 1:**
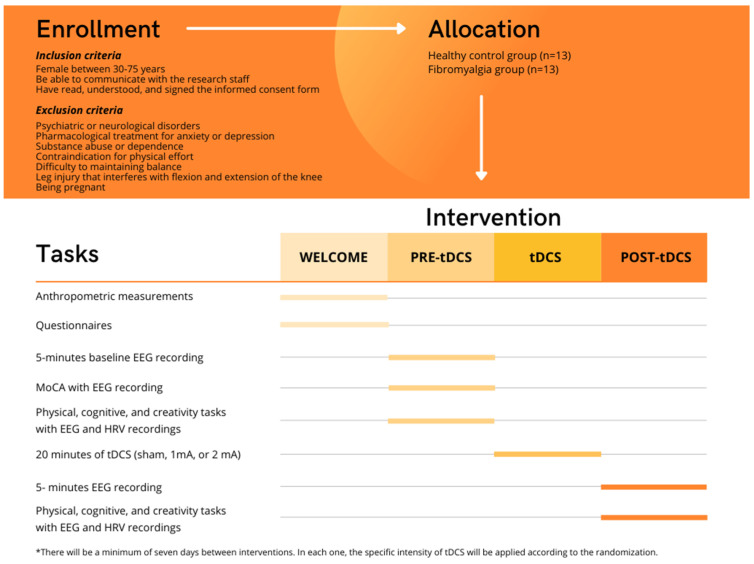
Timeline of the study.

Figure 1 shows the timeline of the study and the course of the intervention. First, an anthropometric measurement will be carried out to calculate the body mass index (BMI) of the participants. Secondly, participants will answer a series of sociodemographic questions and complete the following questionnaires: (1) International Physical Activity Questionnaire (IPAQ), (2) Fibromyalgia Impact Questionnaire-Revised (FIQ-R), (3) Falls Efficacy Scale International (FES-I), (4) EuroQol 5 dimensions-5 levels (EQ-5D-5L) and (5) Pittsburgh Sleep Quality Questionnaire (PSQI).

Pre-tDCS: After completing the questionnaires, a 5 min baseline EEG recording will be performed. This will be followed by the cognitive test (MoCA), during which EEG will also be recorded. This is followed by the physical (balance and strength) and cognitive tasks in single and dual conditions. In this part of the intervention, the creativity tasks will take place between the two physical tests. During the performance of these tests, brain and cardiac electrical activity will be recorded with EEG, and HRV. 

tDCS: The prescribed intensity will be applied in each session for 20 min (sham, 1 mA, and 2 mA). At the end of each tDCS session, the sensations experienced during the stimulation will be assessed using the sensations related to the Transcranial Direct Current Stimulation questionnaire.

Post tDCS: A 5 min EEG recording will be performed. Furthermore, physical, cognitive, and creativity tasks (in this order) will be performed again while EEG and HRV are recorded. 

#### 2.5.2. Primary Outcomes

##### Neurophysiological Variables

Electroencephalography (EEG) and Heart Rate Variability (HRV)

The Enobio^®^ instrument (Neuroelectrics, Cambridge, MA, USA) [61] and Neuroelectrics^®^ instrument driver software (NIC1) will be used to record EEG and HRV signals. The reliability of this device has been proven even when using “dry” electrodes [62]. Therefore, this device allows us to evaluate the EEG signal in 19 channels, according to the International System 10–20, in different brain areas: frontal (Fz, Fp1, Fp2, F3, F4, F7, and F8), central (Cz, C3, and C4), temporal (T3, T4, T5, and T6), parietal (Pz, P3, and P4), and occipital (O1 and O2). Two electrodes placed on the mastoids will act as a reference. The impedance will be kept below 10 KΩ during recording. In this regard, the sampling frequency is 500 Hz, with a 50 Hz notch filter and a bandpass filter from 1 to 40 Hz. In addition, to process data collected in DT, we will use the recommendations provided in the work of Cheron et al. [63] to perform EEG recordings in movement. In this respect, the ASR filter will be used [64]. 

Once the data has been collected, the Matlab toolbox, EEGlab, will be used to preprocess and analyze the data. Artifacts with a noncortical source (eye movements, muscle activity, or line noise) will be corrected by independent component analysis (ICA) [65]. Once all sources of artifacts have been corrected, using the pop_spectopo.m function of EEGlab, we will separate the signal into 6 different spectral power bands: theta (4–7 Hz), alpha-1 (8–10 Hz), alpha-2 (11–12 Hz), beta-1 (13–18 Hz), beta-2 (19–21 Hz), and beta-3 (22–30 Hz).

HRV data will be exported in .edf format to the Kubios HRV software (v. 2.1) [66]. Different HRV variables will be extracted: (a) time domain, such as mean HR (mean HR) RR, the standard deviation of the whole RR interval (SDNN), NN50 count divided by the total number of all NN intervals (Pnn50) and the square root of the mean of the sum of squares of differences between adjacent NN intervals (rMSSD); (b) frequency domain, which includes the ratio of low frequency (LF, 0.04–0.15 Hz)/high frequency (HF, 0.15–0.4 Hz) ratio (LF/HF) and total power; (c) nonlinear measures, such as entropy (ApEn and SampEn). 

##### Physical Fitness

Balance

The tests will be performed on a Kistler force platform (Type 9286a, Kistler Instrumente AG, Winterthur, Switzerland), and the signal recording will be set at 200 Hz [67]. The sway area in cm², the mean amplitude from RMS (root mean square), and the mean velocity in the anteroposterior and mediolateral directions in cm/s will be recorded. In addition, the entropy (SampEn) in the anteroposterior and mediolateral directions will be obtained to determine the regularity with which the COP trajectory repeats [20,67].

Different static posturography tests will be performed to analyze the center of pressure (COP) pattern as well as to establish a sensory analysis of the visual, proprioceptive, and vestibular systems. The tests will be performed in a static position with eyes open (EO) and static position with eyes closed (EC). Each test will have a duration of 45 s [68], where the first 5 s will be considered as adjustment time before starting the recording [69].

Strength

This test will be performed on the isokinetic dynamometer (Multi-Joint 3, Biodex Medical Systems, Inc., Shirley, NY, USA).

Isokinetic dynamometry (ID) allows for the measurement of the strength of the agonist and antagonist muscles at a given angular velocity [70]. From the execution of the test, the peak torque and average peak torque, average power, and total work power will be obtained [70,71,72]. The execution of the test consists of performing a concentric contraction of knee flexion and extension six times [70,72] consecutively and without pause between repetitions at a speed of 60/s and in a range of movement from 0 to 90 degrees, 0 degrees being full extension and 90 degrees being flexion [72]. 

Before the test, 10 squats will be performed as a warm-up. In addition, a familiarization phase with the test will be carried out, in which 10 consecutive repetitions of knee flexion and extension will be performed at a speed of 120/s. Before starting the final test, 1 min of rest time shall be given [73].

Dual-Task Condition

In DT conditions, participants will simultaneously perform the corresponding physical task (strength or balance) and a cognitive task. In this regard, the cognitive task will consist of two-by-two subtractions from a number greater than 100 [74].

Creativity

Firstly, they will be asked to imagine a repetitive situation, and on the other hand, a creative situation [75]: (1) Imagine the reproduction of a model: participants will be asked to imagine themselves reproducing a choreography which has previously shown on a screen (standard and monotonous), and (2) Imagining the performance of a creative task: in the imagined creative task of dance, participants will be asked to perform mentally a free, improvised dance. 

To check that they imagined the requested task, they will have to describe the following aspects verbally after completing the two tasks: (1) imagining reproduction of a model: the color of the room, the elements of the place, the color of the teacher’s clothes, description of movements and a section for them to add further details or observations; (2) imagining the performance of a creative task: the color of the room or place, the elements of the place, description of movements, music or sound, number of stops and a section for them to add further details or observations.

In order to assess brain electrical activity during creative situations, participants will be asked to imagine two different situations with a duration of 3 min each [75].

#### 2.5.3. Secondary Outcomes

##### Sociodemographic Information

Sociodemographic information such as age, level of education, injuries, or falls in the last few months, as well as other health-related information, will be asked.

##### Physical Activity Level

The International Physical Activity Questionnaire (IPAQ) will be used to assess physical activity and inactivity. This has been used to obtain total metabolic equivalents (METs) per week as well as time spent sitting [76]. The Spanish version of the IPAQ [76,77] will be used in this study.

##### Impact of the Disease

Fibromyalgia Impact Questionnaire Revised (FIQ-R)—The FIQ-R [78]—is a self-administered questionnaire consisting of 21 items. Each item is scored from 0 to 10, 10 being the worst condition. It is divided into three domains: (a) function, (b) overall impact, and (c) symptoms, and the maximum score is 100, corresponding to the worst overall symptom impact. The Spanish version of the FIQ-R [79] will be used in this study. This questionnaire will only be administered to people with FM who will participate in this study.

##### Fear of Falling

Falls Efficacy Scale International (FES-I), FES-I, is a self-administered questionnaire that consists of 16 items that are scored on a four-point scale in which the higher the score, the greater the fear of falling [80]. This questionnaire has previously been used in people with FM [81]. The Spanish version of the FES-I will be used in this study [82].

##### Health-Related Quality of Life

EuroQol-5 dimensions-5 levels (EQ-5D-5L) is used to measure health-related quality of life (HRQoL) [83]. It consists of five dimensions (mobility, self-care, activities of daily living, pain or discomfort, and anxiety or depression) and five levels per dimension. The EQ-5D-5L also includes a visual analogue scale (VAS), which rates perceived health status from 0 (worst imaginable health) to 100 (best imaginable health).

##### Sleep Quality

Pittsburgh Sleep Quality Index (PSQI), PSQI, measures the sleep quality and sleep disturbances of the participants over one month on seven components: subjective sleep quality, sleep latency, sleep duration, habitual sleep efficiency, sleep disturbances, use of sleep medication, and daytime dysfunction. Each component is evaluated from 0 to 3, and the total score ranges from 0 to 21 [84]. Higher scores indicate poorer subjective sleep quality. The Spanish version of the PSQI, validated for use in FM patients, will be used [85].

##### Cognitive Impairment

Montreal Cognitive Assessment (MoCA). This brief cognitive screening assesses the following cognitive abilities: attention, concentration, executive functions (including abstraction), memory, language, visual–constructive skills, calculation, and orientation. It has high sensitivity and specificity for detecting mild cognitive impairment even in patients whose performance on the Mini-Mental State Examination (MMSE) is usually in the normal range [86]. It has also been more sensitive than MMSE in people with FM [87]. In addition, 23/30 will be the cut-off point as it has been found to have better diagnostic accuracy, reducing the false positive rate [88]. The administration time required is about ten minutes. The maximum score is 30 [89]. 

##### Sensations Related to tDCS

Survey of sensations related to tDCS. This questionnaire makes it possible to quantify specific subjective feelings and investigate the presence of side or adverse effects of tDCS [90]. A version already published [91] and subsequently modified [92] will be used as a reference. Both versions took into account the effects reported previously [93]. It will be modified, reflecting in each sensation whether it has not been perceived (none) or whether it has been perceived in a mild, moderate, or strong way in each of the sessions received.

##### Anthropometric Measurement 

Body composition will be measured with the Tanita BC-418 Body Composition Analyzer [94]. 

### 2.6. Data Analysis

The outcome results will be included in an anonymous dataset to conduct the statistical analyses. Descriptive and quantitative analyses will be reported. The Statistical Package for the Social Sciences (SPSS, version 24.0; IBM Corp., Armonk, NY, USA) will be employed. Due to the sample size, nonparametric statistical analyses can be the most adequate approach.

In this regard, differences between conditions (1 mA, 2 mA and sham), in all the outcomes, will be analyzed using the Kruskal–Wallis test. Post hoc analyses will be conducted using Mann–Whitney-U tests. Subgroups will be used to study if there are differences in treatment effects according to the presented symptomatology. Furthermore, the [r] effect size will be calculated [95]. Therefore, results will include medians, interquartile ranges, the effect size, and the statistical significance. In order to avoid type I error derived from multiple comparisons, the alpha level of significance will be adjusted according to the Benjamini–Hochberg procedure [96]. 

## 3. Discussion

The present study will be the first to evaluate the effectiveness of tDCS at the PFC in women with FM, as an alternative to reduce cognitive–motor interference in DT conditions, as well as to modify neurophysiological parameters at EEG and autonomic modulation. At the same time, this study will contribute to adding evidence to the existing controversy about which tDCS intensity would be the most effective. Thus, the effects of two common tDCS intensities (1 mA and 2 mA) compared to a simulation (sham) on DT performance and creativity will be analyzed. 

In this study, we first hypothesized that after a tDCS session, the cognitive–motor interference associated with DT performance would decrease in women with FM. In this regard, previous studies have evaluated the effects of tDCS in PFC (offline) on cognitive interference in young adults [27,44,45,46,47,58], healthy older people [48,49] and in Parkinson’s patients [97]. Results of these studies have shown that tDCS could help to reduce cognitive–motor interference during DT performance [27,44,45,46,47,48,49,97]. It is important to highlight that a greater impact on brain activity has been observed in women with FM during DT compared to healthy controls [98]. However, it is still unknown whether the application of tDCS in PFC could reduce the difference in electric brain activation during dual-task conditions between women with FM and healthy controls. 

People with FM showed lower performance when two tasks are conducted at the same time [19,20,21,22]. Lee, Dong, Jeong and Yoon [45] observed that anodal tDCS on the DLPFC can improve memory-guided force control during DT due to altered brain activity. Zhou, Hao, Wang, Jor’dan, Pascual-Leone, Zhang, Fang and Manor [46] showed positive results at the level of postural control and gait. In the same line, Wrightson, Twomey, Ross and Smeeton [27] reported a decrease in stride time variability during gait in DT condition. Furthermore, in older adults, Manor, Zhou, Harrison, Lo, Travison, Hausdorff, Pascual-Leone and Lipsitz [48] showed that tDCS resulted in shorter stride times and less stride time variability in walking while performing DT. Moreover, Zhou, Zhou, Chen, Manor, Lin and Zhang [57] observed that tDCS could increase the complexity of standing postural sway, which is associated with reduced adaptability of the postural control system [57].

There are differences in the stimulation duration and intensity applied in the literature. In this regard, the most common duration was 20 min [44,45,46,48,49,57] and the most common intensity was 2 mA [44,45,48,49,57]. Nevertheless, studies with 1.5 mA also obtained positive results [27,46]. However, few studies have explored the effects of different intensities of tDCS on PFC. Papazova, Strube, Wienert, Henning, Schwippel, Fallgatter, Padberg, Falkai, Plewnia and Hasan [36] did not show differences in the working memory between online tDCS at 1 and 2 mA in the DLPFC. Ehrhardt, Filmer, Wards, Mattingley and Dux [39], who also used online tDCS and stimulation at 0.7, 1 and 2 mA intensities during DT (cognitive–cognitive) training sessions, did not show better DT performance when increasing intensity. Hence, we can hypothesize that in our study, the results after the application of different intensities could be similar. 

Inter- and intra-individual variability make it challenging to find the most appropriate stimulation dose [43,99,100,101]. Thus, there are already studies that propose applying individualized doses to reduce this variability [102,103]. This individual variability has also been studied at the neurophysiological level [104]. In this regard, results showed that the worse the participants’ initial working memory performance was, the more theta power was induced by tDCS in the DLPFC. Thus, we expected that after tDCS in the DLPFC in people with FM, alpha and beta power increased. This would be relevant since a previous study showed that during DT conditions women with FM exhibited lower alpha and beta power compared to healthy controls [98]. 

In addition, different studies have previously discussed how individual variability factors can affect the stimulation response [105,106]. In this line, it is interesting to answer the hypothesis of how the symptoms of fibromyalgia can affect the different effects generated by the treatment. Furthermore, the treatment of these disturbances by tDCS has been studied previously, showing benefits in sleep quality [107], depression [108] and pain [109].

Previous studies have shown that the application of tDCS can induce favorable effects on autonomic modulation in healthy participants [110,111]. Furthermore, Nikolin et al. [112], has shown that after applying tDCS in the DLPFC there was an increase in high frequencies (HF-HRV), which could indicate changes in the parasympathetic branch of the nervous system. This would be relevant since people with FM exhibit an altered autonomic modulation with lower parasympathetic activity and higher sympathetic activity compared to healthy individuals [10]. Thus, we could hypothesize an improvement in the autonomic modulation of women with FM after a tDCS session. 

### 3.1. Future Perspectives in Clinical and Research Issues

Expected results of this study could be extended in future research. Studies with more sessions for each intensity could be conducted to test if similar results are obtained with the same intensity at different times and taking into account the symptomatic variability of fibromyalgia (pain, sleep disturbances or depressive symptoms). In this regard, individual variability in the effects of different intensities of tDCS with this montage on the DLPFC (F3, FP2) to improve performance in DT conditions could be studied.

The beneficial effects obtained following the application of tDCS could be used to complement other clinical interventions, improving the performance of cognitive and/or motor tasks when they must be performed simultaneously, enhancing the effectiveness of these interventions. At the same time, these improvements could help to improve the occupational performance of women with fibromyalgia, and therefore, their quality of life.

### 3.2. Limitations

This study has would have some limitations that should we acknowledge. Firstly, the present study does not report preliminary data, this study only reports the study protocol that will be performed in the future. Secondly, it uses a sample composed of women, so the results will not be generalizable to men suffering from FM. In addition, only the staff involved in performing the stimulation know the intensity applied. Nevertheless, the data analysis will be blinded as it will be performed using code by the investigators who do not perform the stimulation. Furthermore, as there will be only one session of each intensity, targeting only one brain region, we will not be able to be sure whether the observed effects are due to DLPFC stimulation or to more general changes in brain excitability. Finally, since the study will be conducted in the context of the global COVID-19 pandemic, the use of a facemask will be mandatory. On the one hand, cloth or surgical face masks have been shown to have no negative effects on blood or muscle oxygenation and exercise performance and tolerance in healthy individuals [113,114]. Moreover, studies are already proving the safety of the use of face masks in physical exercise [115,116]. However, there are also studies that report negative effects when comparing surgical masks with FFP2/N95, with ventilation, cardiopulmonary exercise capacity and even comfort being more pronounced in the case of FFP2/N95 masks [117]. Furthermore, it has been shown that surgical masks may affect middle-aged people more than younger people [118]. To our knowledge, there are no studies that have reported on the safety or negative effects of mask use in people with FM yet.

## 4. Ethics and Dissemination

The study protocol is currently approved by the Research Ethics Committee of the University of Extremadura (approval number: 192/2021). The data of this research will be confidential, since the participants will sign an informed consent form for the confidentiality of their data. The data obtained from the measurements will only be analyzed by contracted research staff. The results of this study will be published in scientific journals that offer online access, as well as in relevant professional journals. Results will be also presented at national and international conferences.

## 5. Conclusions

This study will be the first to evaluate the effectiveness of tDCS at the PFC in women with FM, as an alternative to reduce cognitive–motor interference in DT conditions, as well as to modify neurophysiological parameters at EEG and autonomic modulation. In this regard, we hypothesized that tDCS would reduce cognitive–motor interference during DT in women with FM. Furthermore, modification of neurophysiological parameters such as HRV or EEG power spectrum (mainly beta and alpha power) are also expected. The study will dilucidate whether the effects differ depending on the stimulation intensity applied (1 mA and 2 mA).

## Data Availability

The data will not be shown publicly, as patients will give their consent for the information to be kept confidential.
